# Sevoflurane postconditioning reduces myocardial ischemia reperfusion injury-induced necroptosis by up-regulation of OGT-mediated O-GlcNAcylated RIPK3

**DOI:** 10.18632/aging.104146

**Published:** 2020-11-20

**Authors:** Jing Zhang, Peng Yu, Fuzhou Hua, Yanhui Hu, Fan Xiao, Qin Liu, Dan Huang, Fumou Deng, Gen Wei, Wei Deng, Jianyong Ma, Wengen Zhu, Jiru Zhang, Shuchun Yu

**Affiliations:** 1 Department of Anesthesiology, The Second Affiliate Hospital of Nanchang University, Nanchang 330006, China; 2 Department of Endocrinology and Metabolism, The Second Affiliate Hospital of Nanchang University, Nanchang 330006, China; 3 Department of Pharmacology and Systems Physiology, University of Cincinnati College of Medicine, Cincinnati, OH 45267, USA; 4 Department of Cardiology, The First Affiliated Hospital of Sun Yat-Sen University, Guangzhou 510080, Guangdong, China; 5 Department of Anesthesiology, Affiliated Hospital of Jiangnan University (The Fourth People's Hospital in Wuxi City), Wuxi 214000, China

**Keywords:** sevoflurane postconditioning, myocardial ischemia reperfusion injury, OGT, O-GlcNAcylation, necroptosis

## Abstract

Inhalation anesthetics have been demonstrated to have protective effects against myocardial ischemia reperfusion injury (MIRI). O-linked GlcNAcylation (O-GlcNAc) modifications have been shown to protect against MIRI. This study aimed to investigate whether O-GlcNAcylation and necroptosis signaling were important for sevoflurane postconditioning (SPC) induced cardioprotective effects. Apart from rats in the SHAM and sevoflurane (SEVO) group, rats underwent 30 min ischemia followed by 2 h reperfusion. Cardiac hemodynamics and function were determined. In addition, myocardial infarction size, cardiac function parameters, myocardial lactic dehydrogenase (LDH) content, myocardium histopathological changes, necrotic myocardium, O-GlcNAcylation, and protein expression levels of necroptosis biomarkers were measured, together with co-immunoprecipitation experiments using proteins associated with the necroptosis pathway and O-GlcNAcylation. SPC reduced myocardial infarction size, ameliorated cardiac function, restored hemodynamic performance, improved histopathological changes, and reduced receptor-interacting protein kinase 1 (RIPK1)/receptor-interacting protein kinase 3 (RIPK3)/mixed lineage kinase domain-like (MLKL) mediated necroptosis. In addition, SPC up-regulated O-GlcNAc transferase (OGT) mediated O-GlcNAcylation, increased O-GlcNAcylated RIPK3, and inhibited the association of RIPK3 and MLKL. However, OSMI-1, an OGT inhibitor, abolished SPC mediated cardioprotective effects and inhibited OGT mediated up-regulation of O-GlcNAcylation and down-regulation of RIPK3 and MLKL proteins induced by SPC. Our study demonstrated that SPC restrained MIRI induced necroptosis via regulating OGT mediated O-GlcNAcylation of RIPK3 and lessening the formulation of RIPK3/MLKL complex.

## INTRODUCTION

Myocardial ischemia reperfusion injury (MIRI) is central to the pathology of major cardiovascular diseases, such as stroke and myocardial infarction. Developing effective treatment strategies to protect the myocardium against ischemia reperfusion injury is critically important [1]. MIRI is a common complication of ischemic heart disease. It refers to further aggravation of myocardium injury due to blood perfusion after ischemia myocardial injury.

Sevoflurane is a volatile anesthetic widely used in cardiovascular surgery. It has a low blood-gas distribution index, rapid induction and recovery after drug withdrawal, and a slight effect on circular respiration [2]. Our previous studies demonstrated that sevoflurane postconditioning (SPC) had protective effects against MIRI, and the cardioprotective effects were modulated by multiple signaling pathways [3–4]. The potential cardioprotective effects of SPC has been demonstrated in many clinical and laboratory studies. However, the complex molecular mechanisms of SPC have not been fully elucidated.

Previous studies have found that ischemic preconditioning and postconditioning reduced cell necroptosis induced by ischemia reperfusion injury [5]. Necroptosis, a form of cell death resembling a necrotic phenotype, has been identified in cardiac pathologies. Its inhibition has been demonstrated to be cardioprotective. Previous studies have demonstrated that ischemic preconditioning protects against ischemia reperfusion injury by inhibiting receptor-interacting protein kinase 1 (RIPK1)/receptor-interacting protein kinase 3 (RIPK3)/mixed lineage kinase domain-like (MLKL) axis mediated necroptosis [6]. Necroptosis is mediated by the necrosome, a signaling protein complex that is composed of RIPK3 and MLKL [7]. Recent studies have shown that O-GlcNAc transferase (OGT) suppresses necroptosis by targeting RIPK3 [8].

O-linked GlcNAcylation (O-GlcNAc), a dynamic and reversible posttranslational modification, regulates diverse cellular processes, including translation, transcription, protein trafficking, stress responses, and metabolic signaling [9, 10]. The cycling of O-GlcNAc modifications is actively controlled by a pair of enzymes, which consist of O-GlcNAc transferase (OGT) and O-GlcNAcase (OGA). RIPK3 has been demonstrated to be an important regulatory protein for necroptosis, while OGT serves as a negative regulator of necroptosis by inhibiting RIPK3 expression [11, 12]. Previous studies have shown that dysfunction in O-GlcNAcylation sensitizes cells to various types of injury and stress, eventually leading to cell necroptosis [13, 14]. Previous studies have also demonstrated that higher expression of O-GlcNAc modified proteins in myocardia had anesthetic preconditioning mediated cardioprotective effects on MIRI [15]. However, it is unknown whether OGT mediated O-GlcNAcylation and necroptosis signaling are involved in SPC mediated protective effects on MIRI. The potential molecular mechanisms underlying SPC protective effects are complex and are yet to be deciphered.

We hypothesized that SPC reduces MIRI induced necroptosis, and OGT mediated O-GlcNAcylation plays a significant role. Hence, in the present study, we investigated the cardioprotective effects and mechanism of SPC by evaluating OGT mediated O-GlcNAcylation and necroptosis signaling using an *in vivo* ischemia reperfusion model and Langendorff isolated heart perfusion model.

## RESULTS

### SPC reduces myocardial infarction size, improves cardiac function and hemodynamic performance, and attenuates histopathological changes

**Figure 1:**
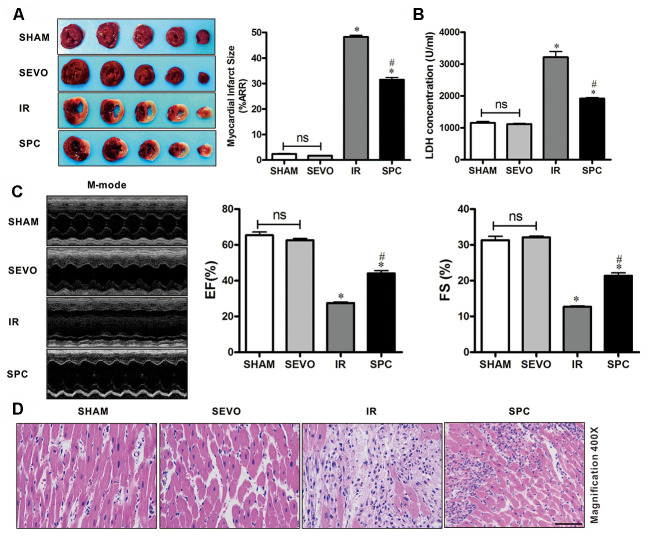
**SPC reduced myocardial infarction size, recovered cardiac function, decreased LDH level and attenuated histopathological changes.** (**A**) Myocardial infarct size was measured by 1% TTC and expressed as a percentage of area at risk. n=6/group. (**B**) Myocardial necroptosis marker enzyme LDH was determined by commercially available LDH kits. n=6/group. (**C**) Representative M-mode images of echocardiography and EF%, FS% were recorded in all groups. n=8/group. (**D**) Representative H&E staining images are shown (magnification, 400×). Scale bar, 50μm. n = 3/group. The columns and errors bars represent means ± SD. * P < 0.05 vs. SHAM group; # P < 0.05 vs. IR group.

To demonstrate the protective effects of SPC on MIRI, we measured myocardial infarction size using 1% TTC, the levels of serum cardiac necroptosis marker enzyme LDH, cardiac function by echocardiography, and histopathological changes by H&E staining. Consistent with previous studies [3], we observed that 30 min of LAD occlusion induced significant myocardial infarction in the IR group (48.26±1.61% of the total area). As shown in Figure 1A, treatment with 1.0 MAC sevoflurane during the first 15 min of reperfusion reduced infarct size to 31.51±2.09% in the SPC group. Compared to the IR group, the infarction area in the SPC group was significantly reduced (*P*<0.05, Figure 1A). However, infarction areas in the SHAM and SEVO group were not statistically different (*P*>0.05, Figure 1A).

Myocardial damage was determined by measuring LDH release into the coronary effluent. MIRI increased the level of serum cardiac necroptosis marker enzyme LDH in the IR group, while SPC administration attenuated the increase in LDH levels in the SPC group (*P*<0.05, Figure 1B). No statistical differences were observed in LDH levels between the SHAM and SEVO group (*P*>0.05, Figure 1B).

We next examined impaired left ventricle (LV) systolic function by echocardiography. As shown in Figure 1C and Table 1, there were no significant differences in LV contractility between the SHAM and SEVO group (*P*>0.05). Compared to the SHAM group, the systolic function was dramatically reduced in the IR group, with a significant decrease in EF%, FS%, and SV (*P*<0.05, Figure 1C, Table 1). Indicators of cardiac remodeling were reduced. Interventricular septal thickness at diastolic phase (IVSd), interventricular septal thickness at systolic phase (IVSs), left ventricular posterior wall thickness at diastolic phase (LVPWd) and left ventricular posterior wall thickness at systolic phase (LVPWs) were significantly reduced, while left ventricular internal diameter at diastolic phase (LVIDd) and left ventricular internal diameter at systolic phase (LVIDs) levels were significantly increased (*P*<0.05, Table 1). After the administration of SPC, LV systolic function was improved in the SPC group. The decrease in EF%, FS%, and SV induced by MIRI were inhibited after SPC administration in the SPC group (*P*<0.05, Figure 1C, Table 1). Compared to the IR group, indicators of cardiac remodeling were improved with an increase in IVSd, IVSs, LVPWd, LVPWs, and a decrease in LVIDd and LVIDs in the SPC group (*P*<0.05, Figure 1C, Table 1).

**Table 1:** Cardiac function measured by echocardiography.

**Cardiac function**	**SHAM (*n* = 8)**	**SEVO (*n* = 8)**	**IR (*n* = 8)**	**SPC (*n* = 8)**
EF (%)	64.76±1.58	65.28±1.89	31.07±1.49^*^	46.56±4.34^*#^
FS (%)	33.91±2.05	34.98±2.34	15.98±2.12^*^	23.30±4.31^*#^
SV (ml)	152.16±7.58	156.27±9.53	100.28±9.66^*^	126.58±12.01^*#^
IVSd (mm)	1.44±0.13	1.51±0.11	1.07±0.12^*^	1.31±0.10^*#^
IVSs (mm)	2.26±0.20	2.38±0.34	1.31±0.13^*^	1.95±0.20^*#^
LVPWd (mm)	1.76±0.24	1.82±0.24	1.24±0.08^*^	1.48±0.11^*#^
LVPWs (mm)	2.31±0.26	2.28±0.16	1.48±0.13^*^	1.81±0.09^*#^
LVIDd (mm)	6.61±0.27	6.47±0.44	8.50±0.33^*^	7.48±0.15^*#^
LVIDs (mm)	4.60±0.21	4.35±0.30	6.36±0.30^*^	5.54±0.23^*#^

* P <0.05 vs SHAM group; # *P* <0.05 vs IR group. Values are means ± standard deviation. n = 8 /group. EF, ejection fraction; FS, fractional shortening; SV, stroke volume; IVSd, interventricular septal thickness at diastolic phase; IVSs, interventricular septal thickness at systolic phase; LVPWd, left ventricular posterior wall thickness at diastolic phase; LVPWs, left ventricular posterior wall thickness at systolic phase; LVIDd, left ventricular internal diameter at diastolic phase; LVIDs, left ventricular internal diameter at systolic phase. SHAM, sham control; SEVO, sevoflurane alone; IR, ischemia reperfusion; SPC, sevoflurane postconditioning.

Our study also evaluated hemodynamic performance. Hemodynamic parameters included HR, MAP, and RPP. The *in vivo* hemodynamics at baseline showed no statistical differences for all the groups (*P*>0.05, Table 2). At T_2_, T_3_ and T_4_, HR, MAP, and RPP decreased in the IR and SPC group (*P*<0.05 vs T_0_, Table 2). Ischemia reperfusion significantly deteriorated hemodynamic performance with a decrease in HR, MAP, and RPP (*P*<0.05 vs SHAM group, Table 2). SPC significantly improved hemodynamic injury indexes induced by MIRI during reperfusion (*P*<0.05 vs IR group, Table 2).

**Table 2:** Hemodynamics *in vivo* experiments.

	**Group**	**T_0_**	**T_1_**	**T_2_**	**T_3_**	**T_4_**
**HR (min^-1^)**	**SHAM**	270 ± 22	268 ± 14	259 ± 21	251 ± 10	248 ± 16
	**SEVO**	275 ± 12	267 ± 14	262 ± 14	261 ± 16	258 ± 25
	**IR**	268 ± 19	254 ± 23	206 ± 10^*#^	156 ± 28^*#^	128 ± 12^*#^
	**SPC**	258 ± 18	251 ± 25	211 ± 24^*#^	191 ± 21^*#&^	182 ± 16^*#&^
**MAP (mmHg)**	**SHAM**	123 ± 17	112 ± 15	111 ± 7	108 ± 11	107 ± 8
	**SEVO**	121 ± 11	113 ± 11	109 ± 7	109 ± 11	108 ± 10
	**IR**	114 ± 11	100 ±19	67 ±5^*#^	52 ± 3^*#^	47 ± 3^*#^
	**SPC**	114 ± 4	96 ± 9	89 ± 10^*#^	84 ± 10^*#&^	75 ± 7^*#&^
**RPP (min^-1^mmHg ×10^3^)**	**SHAM**	47 ± 11	46 ± 6	43 ± 3	40 ± 4	38 ± 6
	**SEVO**	47 ± 7	44 ± 5	44 ± 8	43 ± 6	40 ± 17
	**IR**	46 ± 3	41 ±8	30 ±6^*#^	21 ± 6^*#^	19 ± 3^*#^
	**SPC**	45 ± 7	41 ± 4	34 ± 3^*#^	29 ± 4^*#&^	28 ± 3^*#&^

* *P*<0.05 vs T_0_; # *P*<0.05 vs SHAM group; &*P*<0.05 vs IR group. Values are means ± standard deviation. n = 6 /group. HR, heart rate; MAP, mean arterial blood pressure; RPP, rate pressure product. T_0_, equilibration; T_1_, 30 min after reperfusion; T_2_, 60 min after reperfusion; T_3_, 90 min after reperfusion; T_4_, 2h after reperfusion. SHAM, sham control; SEVO, sevoflurane alone; IR, ischemia reperfusion; SPC, sevoflurane postconditioning.

Myocardial histopathological changes were evaluated in H&E-stained heart sections. As shown in Figure 1D, H&E stained sections demonstrated that the hearts from the SHAM and SEVO group had myocardial structures arranged regularly, normal-sized cardiomyocytes with clear boundaries, and arranged regularly. Compared to the SHAM group, rat hearts from the IR group had loosely and irregularly arranged, of which, the outlines were difficult to identify. In addition, the myocardium had unclear or disordered transverse striations, intracytoplasmic vacuoles, and edema in the cardiomyocytes. However, compared to the IR group, myocardium injury in the SPC group was significantly reduced. Rat hearts in the SPC group had markedly decreased percentage of aberrant and irregular myocardium, relatively clear and well-distributed transverse striations, and cardiomyocytes (Figure 1D). These results demonstrate that SPC alleviated MIRI induced myocardial injury and improved cardiac function in the ischemia reperfusion rat model.

### SPC reduces MIRI-induced necroptosis and the expression of RIPK3 and MLKL

**Figure 2:**
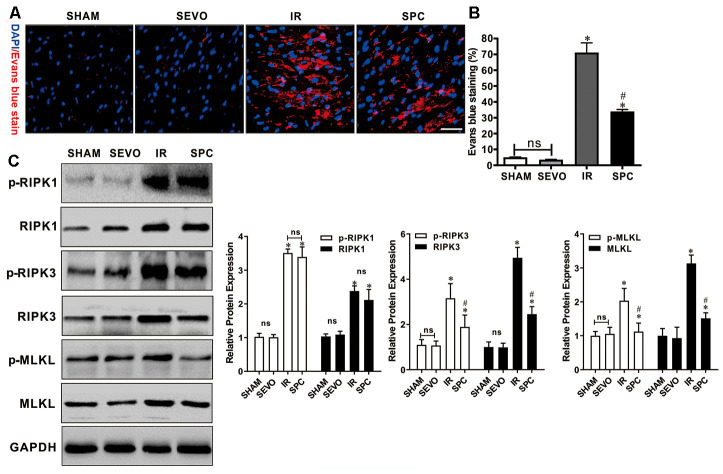
**SPC inhibited necroptosis and the up-regulation of RIPK3, MLKL proteins induced by MIRI.** (**A**) Analysis of IR injury induced necroptosis via Evans blue dye uptake in the hearts. (**B**)The percent area of EBD positive myocardium is shown. n=3/group. (**C**) Immunoblotting was used to examine the expression levels of p-RIPK1, RIPK1, p-RIPK3, RIPK3, p-MLKL and MLKL proteins. Representative protein images and quantitative analysis were shown. n = 3 /group. The columns and errors bars represent means ± SD. * *P* < 0.05 vs. SHAM group; # *P* < 0.05 vs. IR group.

Evans blue dye uptake, an indicator for detecting necroptosis [16], was markedly increased after IR injury. Evans blue staining was used to determine the effect of SPC on necroptosis induced by MIRI. Compared to the IR group, SPC administration significantly reduced Evans blue dye uptake in the SPC group (*P*<0.05, Figure 2A, 2B). We then measured the protein expression levels of p-RIPK1, RIPK1, p-RIPK3, RIPK3, p-MLKL, and MLKL, which are important regulatory proteins for necroptosis. Western blotting demonstrated a significant increase in protein expression for the above regulatory proteins in the IR and SPC group compared to the SHAM group (*P*<0.05, Figure 2C). Compared to the IR group, SPC inhibited the up-regulation of p-RIPK3, RIPK3, p-MLKL, and MLKL protein expression (*P*<0.05, Figure 2C). The protein expression levels of p-RIPK3 and RIPK3 in SPC group were down-regulated significantly compared with the IR group, the reduction ratio of p-RIPK3 and RIPK3 was 40.10% and 50.34% respectively (*P*<0.05, Figure 2C). MLKL, as a downstream of RIPK3, promoted the occurrence of necroptosis. The Figure 2C, 2D also showed that the protein expression levels of p-MLKL and MLKL in SPC group was 40.10% and 40.10% decreased respectively compared with the IR group. However, the expression levels of p-RIPK1 and RIPK1 in the IR group and SPC group had no statistical differences (*P*>0.05, Figure 2C). Collectively, our results indicate that SPC administration suppressed the up-regulation of RIPK3/MLKL mediated necroptosis induced by MIRI.

### SPC up-regulates OGT mediated O-GlcNAcylation

**Figure 3:**
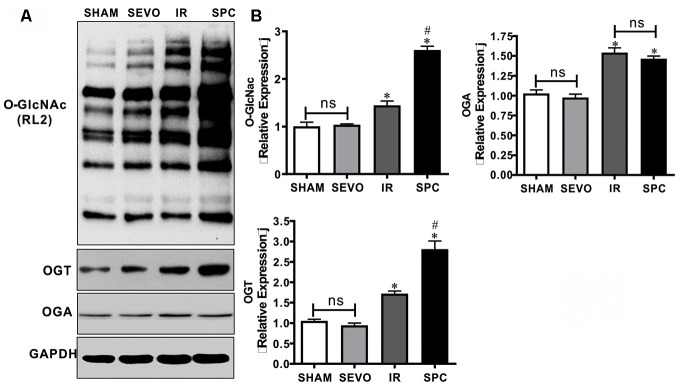
**SPC up-regulated the expression of O-GlcNAc and OGT proteins.** (**A**) Representative protein images of O-GlcNAc, OGT and OGA from all groups were shown. (**B**) Quantitative analysis of O-GlcNAc, OGT and OGA proteins were performed. n = 3 /group. The columns and errors bars represent means ± SD. * P < 0.05 vs. SHAM group; # P < 0.05 vs. IR group.

OGT has been demonstrated to act as a negative regulator of necroptosis by inhibiting RIPK3 expression, while O-GlcNAcylation dysfunction sensitizes cells to various kinds of stress, which ultimately results in cell necroptosis [13, 14, 17]. Hence, we measured the expression levels of O-GlcNAc, OGT and OGA to determine the underlying mechanism of SPC. OGT and OGA act as a pair of enzymes that actively control O-GlcNAc modification cycling. Western blot analysis demonstrated that O-GlcNAc, OGT and OGA protein expression levels in the IR and SPC groups were increased compared to the SHAM and SEVO groups (*P*<0.05, Figure 3). SPC administration further up-regulated the expression levels of O-GlcNAc and OGT in the SPC group (*P*<0.05, Figure 3). However, no significant differences in OGA protein expression levels were observed between the IR and SPC groups (*P*>0.05, Figure 3). This indicated that SPC enhances OGT mediated O-GlcNAcylation.

### SPC increases O-GlcNAcylated RIPK3 and inhibits the binding of RIPK3 and MLKL

**Figure 4:**
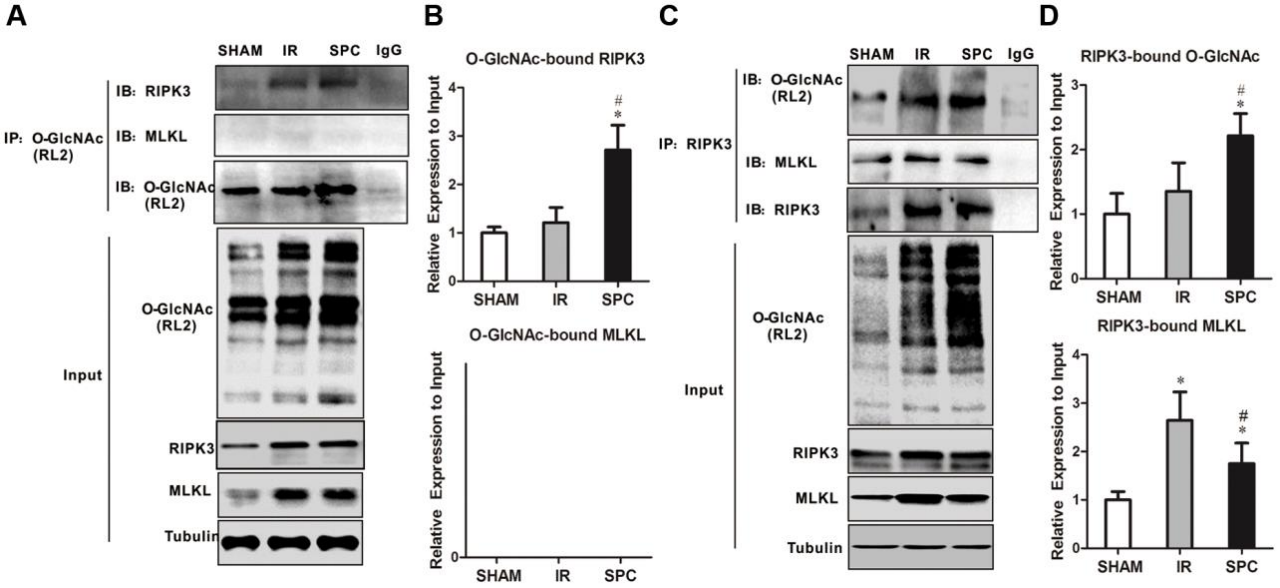
**SPC increased O-GlcNAcylated RIPK3 and inhibited the combination of RIPK3 and MLKL.** (**A**) RIPK3 and MLKL were detected in O-GlcNAc immunoprecipitates. Representative protein images were performed. (**B**) Quantitative analysis of O-GlcNAcylated RIPK3 and MLKL. n = 3 /group. (**C**) O-GlcNAc and MLKL in RIPK3 immunoprecipitates. Representative protein images were shown. (**D**) Quantitative analysis of O-GlcNAcylated RIPK3 and the complex between RIPK3 with MLKL. n = 3 /group.

RIPK3 has been demonstrated to be a target protein for O-GlcNAc modification. To identify potential mechanisms on how SPC enhances O-GlcNAcylation to regulate necroptosis, we performed reciprocal immunoprecipitation and western blot assays. As shown in Figure 4A, 4B, RIPK3 co-immunoprecipitated with RL2, which was consistent with previous studies [8, 12]. However, MLKL was not detected in RL2 immunoprecipitates (Figure 4A, 4B). Moreover, rat hearts from the SPC group had an increased ratio of O-GlcNAc-modified RIPK3 compared to the SHAM and IR groups (Figure 4A, 4B). We then performed anti-RIPK3 antibody immunoprecipitations. As shown in Figure 4C, 4D, both O-GlcNAc and MLKL were detected in RIPK3 immunoprecipitates. Relatively higher levels of O-GlcNAcylated RIPK3 were observed in rat hearts from the SPC group compared to the other groups. RIPK3-MLKL complex formation in the heart was reduced after SPC administration (Figure 4C, 4D). These results indicate that SPC promotes OGT-mediated O-GlcNAcylation of RIPK3, reduces the expression of RIPK3, and eventually lead to reduction in the RIPK3-MLKL complex formation.

### Pharmacological inhibition of OGT abrogates the protective effects of SPC against MIRI in isolated hearts

**Figure 5:**
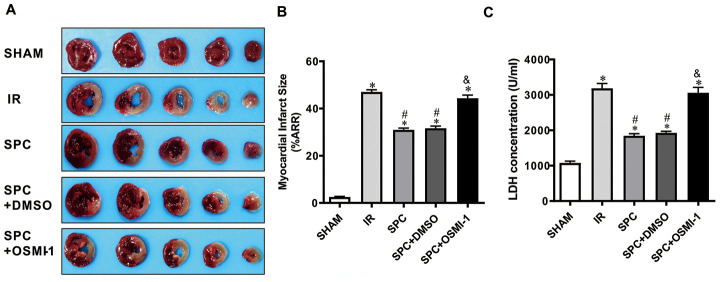
**OSMI-1 eliminated SPC-mediated decreases in cardiac infarction and the LDH level.** (**A**) Infarct size was determined by using 1%TTC staining. Represetnative images were shown. (**B**) Infarct size was expressed as a percentage of area at risk. n=6/group. (**C**) Myocardial necroptosis marker enzyme LDH level in all groups. OSMI-1, the OGT inhibitor; DMSO, the OSMI-1 solvent. n=6/group. The columns and errors bars represent means ± SD. * P < 0.05 vs. SHAM group; # P < 0.05 vs. IR group; & P < 0.05 vs. SPC group.

We demonstrated that SPC induced cardioprotection may be associated with OGT mediated O-GlcNAcylation. We then made use of the Langendorff isolated heart perfusion model to validate the direct relation between OGT mediated O-GlcNAcylation and SPC cardioprotective effects. OSMI-1, an OGT inhibitor, has been demonstrated to reduce global protein O-GlcNAcylation levels [18]. As shown in Figure 5A, 5B, MIRI dramatically increased rat heart infarct size (2.53±0.52%vs.46.98±2.32%, *P*<0.05), while SPC administration inhibited the increase in myocardial infarct size in the SPC group (*P*<0.05, Figure 5A, 5B). However, after the administration of OGT inhibitor, the myocardial infarct size was significantly increased in the SPC+OSMI-1 group compared to the SPC group (44.35±3.34% vs. 30.96±1.81%, *P*<0.05, Figure 5A, 5B). No statistical difference in myocardial infarct size was observed between the SPC and SPC+DMSO group (*P*>0.05, Figure 5A, 5B).

Hemodynamic differences for all the experimental groups had the same trend. Hemodynamics at baseline (T_0_) for the five groups showed no significant differences (*P*>0.05, Table 3). Compared to the equilibrium phase in the SHAM group, hemodynamic indexes were exacerbated with the decrease in HR, LVSP, ±dp/dt_max_ and the increase in LVEDP during the ischemia reperfusion periods (T_1_, T_2_, T_3,_ and T_4_) in the remaining groups (*P*<0.05, Table 3). As expected, SPC administration significantly reduced the decline in hemodynamic indexes induced by MIRI (*P*<0.05, Table 3). More remarkably, compared to the SPC group, the OGT inhibitor OSMI-1 significantly restrained HR, LVSP, ±dp/dt_max,_ and enhanced LVEDP in the SPC+OSMI-1 group (*P*<0.05, Table 3).

**Table 3:** Hemodynamics *in vitro* experiments.

	**Group**	**T_0_**	**T_1_**	**T_2_**	**T_3_**	**T_4_**
**HR (min^-1^)**	SHAM	262 ± 16	250 ± 22	248 ± 10	246 ± 22	235 ± 32
	IR	258 ± 17	198 ± 14^*#^	173 ± 12^*#^	150 ± 9^*#^	124 ± 12^*#^
	SPC	260 ± 11	213 ± 7^*#&^	198 ± 10^*#&^	180 ± 18^*#&^	156 ± 19^*#&^
	SPC+DMSO	264 ± 21	211 ± 6^*#&^	193 ± 11^*#&^	180 ± 7^*#&^	157 ± 18^*#&^
	SPC+OSMI-1	265 ± 20	205 ± 9^*#^	158 ± 12^*#^	144 ± 15^*#^	137 ± 13^*#^
**LVSP (mmHg)**	SHAM	110 ± 5	108 ± 7	102 ± 10	96 ± 5	94 ± 9
	IR	115 ± 12	64± 6^*#^	48 ± 6^*#^	44 ± 4^*#^	35 ± 4^*#^
	SPC	106 ± 18	93 ±5^*#&^	81 ±6^*#&^	64 ± 4^*#&^	59 ± 4^*#&^
	SPC+DMSO	110 ± 11	91 ± 6^*#&^	85 ± 4^*#&^	66 ± 6^*#&^	58 ± 6^*#&^
	SPC+OSMI-1	109 ± 12	69 ± 4^*#^	53 ± 7^*#^	45 ± 7^*#^	35 ± 4^*#^
**LVEDP (mmHg)**	SHAM	6.7 ± 0.8	7.2 ±0.6	7.3 ± 0.5	7.4 ± 0.5	7.6 ± 1.1
	IR	7.0 ± 0.2	37.6 ± 2.3^*#^	42.1 ± 3.7^*#^	47.3 ± 1.9^*#^	51.7 ± 4.2^*#^
	SPC	7.4 ± 0.6	21.6 ± 1.9^*#&^	25.9 ± 2.1^*#&^	29.2 ± 1.1^*#&^	31.6 ± 1.9^*#&^
	SPC+DMSO	7.0 ± 0.4	23.5 ± 1.5^*#&^	27.7 ± 3.4^*#&^	29.8 ± 0.8^*#&^	31.7 ± 2.1^*#&^
	SPC+OSMI-1	6.6 ± 0.6	38.5 ± 2.7^*#^	42.1 ± 4.5^*#^	45.0 ± 1.8^*#^	51.4 ± 5.8^*#^
**+dp/dt_max_ (mmHg/s)**	SHAM	2330 ± 218	2272 ±203	2159 ± 92	2008 ± 192	1924 ± 158
	IR	2188 ± 183	1853 ± 174^*#^	1586 ± 93^*#^	1358 ± 157^*#^	1145 ± 79^*#^
	SPC	2281 ± 239	2160 ± 164^*#&^	2067 ± 202^*#&^	1786 ± 156^*#&^	1457 ± 107^*#&^
	SPC+DMSO	2293 ± 261	2078 ± 185^*#&^	2001 ± 169^*#&^	1748 ± 234^*#&^	1542 ± 144^*#&^
	SPC+OSMI-1	2324 ± 191	1927 ± 115^*#^	1721 ± 145^*#^	1383 ± 158^*#^	1141 ± 131^*#^
**-dp/dt_max_ (mmHg/s)**	SHAM	2306 ± 228	2215 ±218	2158 ± 140	2087 ± 184	2053 ± 219
	IR	2335 ± 213	1912 ± 89^*#^	1418 ± 175^*#^	1096 ± 72^*#^	888 ± 64^*#^
	SPC	2322 ± 173	2078 ± 301^*#&^	1865 ± 87^*#&^	1435 ± 159^*#&^	1103 ± 99^*#&^
	SPC+DMSO	2269 ± 137	2047 ± 139^*#&^	1796 ± 199^*#&^	1450 ± 111^*#&^	1116 ± 77^*#&^
	SPC+OSMI-1	2376 ± 133	1864 ± 217^*#^	1557 ± 228^*#^	1024 ± 80^*#^	901 ± 101^*#^

*P < 0.05 vs T0; # *P* < 0.05 vs SHAM group; & *P* < 0.05 vs IR group. Values are means ± standard deviation. n = 6 /group. HR, heart rate; LVSP, left ventricular peak pressure; LVEDP, left ventricular end diastolic pressure; +dp/dt_max_, maximal rate of the increase of left ventricular pressure; -dp/dt_max_, maximal rate of the decrease of left ventricular pressure. T_0_, equilibration; T_1_, 30 min after reperfusion; T_2_, 60 min after reperfusion; T_3_, 90 min after reperfusion; T_4_, 2h after reperfusion. SHAM, sham control; IR, ischemia reperfusion; SPC, sevoflurane postconditioning; OSMI-1, the OGT inhibitor; DMSO, the OSMI-1 solvent.

LDH levels were measured using an *in vitro* model. MIRI up-regulated LDH levels in the IR group, while SPC inhibited the increase in LDH levels induced by MIRI (*P*>0.05, Figure 5C). After OSMI-1 administration, LDH levels in the SPC+OSMI-1 group were higher compared to the SPC group (*P*>0.05, Figure 5C). In line with our *in vivo* studies, SPC could significantly reduce myocardial infarction induced by MIRI and had cardioprotective effects against IR injury. However, pharmacological inhibition of OGT using OSMI-1 eliminated SPC induced protective effects on MIRI in isolated rat hearts.

### Pharmacological inhibition of OGT abrogates the up-regulation of O-GlcNAcylation and down-regulation of necroptosis signaling induced by SPC

**Figure 6:**
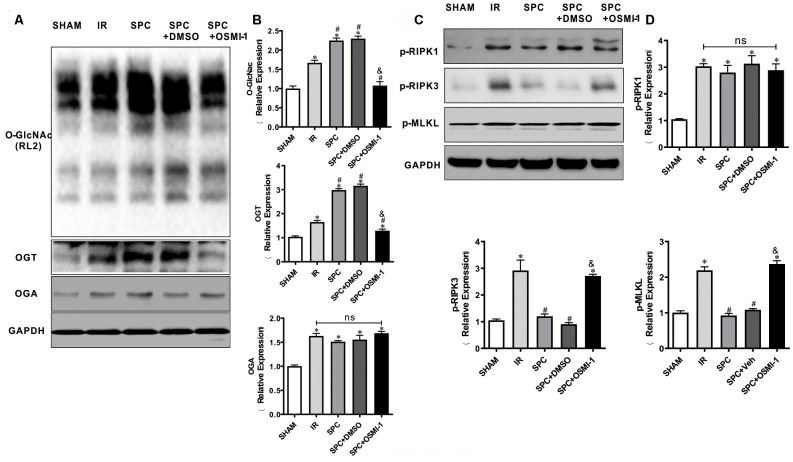
**OSMI-1 abolished SPC induced activation in OGT mediated O-GlcNAcylation and reduction in p-RIPK3, p-MLKL.** (**A**, **B**) Expressions of O-GlcNAc, OGT and OGA in all groups were analyzed by Western Blot. Representative protein images and quantitative analysis were shown. n=3/group. (**C**, **D**) Representative protein images and quantitative analysis of p-RIPK1, p-RIPK3, and p-MLKL in all groups were presented. n=3/group. OSMI-1, the OGT inhibitor; DMSO, the OSMI-1 solvent. The columns and errors bars represent means ± SD. * P < 0.05 vs. SHAM group; # P < 0.05 vs. IR group; & P < 0.05 vs. SPC group.

We next investigated the effect of OSMI-1 administration on OGT mediated O-GlcNAcylation and necroptosis signaling. Similar to our *in vivo* results, SPC additionally up-regulated OGT mediated O-GlcNAcylation (*P*<0.05 vs SHAM group) (Figure 6A, 6B). However, compared to the SPC group, pharmacological inhibition of OGT could down-regulate OGT mediated O-GlcNAcylation (*P*<0.05, Figure 6A, 6B). Based on the direct measurement of myocardial infract size and LDH levels, these results indicated that OGT mediated O-GlcNAcylation is mainly involved in SPC mediated protection in I/R hearts.

Western blot analysis demonstrated that SPC down-regulated p-RIPK3 and p-MLKL expression levels in our *in vitro* model (*P*<0.05 vs SHAM group, Figure 6C, 6D), while OSMI-1 reversed the down-regulation of p-RIPK3 and p-MLKL induced by SPC (*P*<0.05, Figure 6C, 6D). Our results demonstrated that OSMI-1 inhibits OGT mediated O-GlcNAcylation and up-regulates necroptosis signaling to reverse the protective effects of SPC.

**Figure 7:**
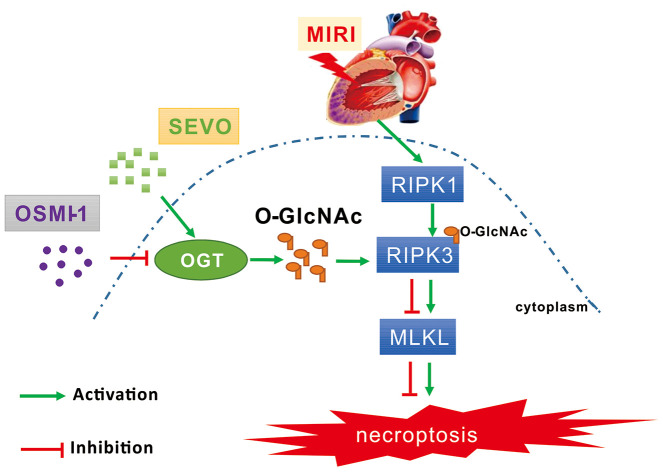
**Schematic diagram depicting the role of OGT mediated O-GlcNAcylation and necroptosis signaling in the SPC induced cardioprotective effect on MIRI. MIRI could induce the up-regulation of RIPK1/RIPK3/MLKL axis mediated necroptosis.** And SPC promoted the increase of OGT induced O-GlcNAcylation, enhanced the RIPK3 O-GlcNAcylation, and then inhibited the formation of the RIPK3/MLKL complex, finally lead to the inhibition of MIRI mediated necroptosis. Furthermore, the protective effects of SPC against MIRI was abrogated by using the OGT inhibitor OSMI-1. In brief, SPC restrained cardiomyocytes necroptosis via regulating OGT mediated O-GlcNAcylation of RIPK3 and lessening RIPK3/MLKL induced necroptosis, and hence protected the heart against MIRI.

## DISCUSSION

We demonstrated, for the first time, that SPC administration increased RIPK3 protein O-GlcNAc levels in an OGT dependent manner and that augmentation of this response was associated with reduced myocardial infarction size, restoration of cardiac function, and decreased necroptosis following simulated ischemia reperfusion injury *in vivo* and *in vitro*. Furthermore, we demonstrated that OSMI-1, a putative OGT inhibitor, markedly reduced O-GlcNAc levels and the interaction between RIPK3 and O-GlcNAclation, which eventually lead to increased expression of p-RIPK3 and p-MLKL. In addition, SPC mediated cardioprotective effects were abrogated after pharmacological inhibition of OGT.

Sevoflurane is an anesthetic of excellent quality, possesses little systemic toxicity, undergoes limited biotransformation, and plays an important role in reducing MIRI. Numerous clinical and laboratory studies have demonstrated the protective effects and associated mechanisms of SPC on MIRI [19]. Our previous study demonstrated that SPC protected against MIRI. Activation of the phosphatidylinositol-3-hydroxykinase (PI3K)/protein kinase B (AKT)/mammalian target of rapamycin (mTOR) and extracellular signal-regulated kinase (ERK)1/2 pathway, inhibition of the toll-like receptor 4 (TLR4)/myeloid differential protein 88 (MyD88)/nuclear factor kappa-B (NF-κB) signaling pathway, restoring mitochondrial function, reducing oxidative stress and restoring autophagic clearance were important mechanisms of this cardioprotective effects [3, 4, 20, 21]. In the present study, we demonstrated that SPC reduced myocardial infarction size, restored cardiac function, improved hemodynamic performance, attenuated histopathological changes, and induced cardioprotective effects on MIRI (Figure 1).

Furthermore, recent studies have demonstrated that SPC induced protective effects were not only limited to the heart but was also observed in multiple organs, such as the brain, liver, lungs, and kidneys [22–26]. SPC has also been shown to alleviate hepatic ischemia reperfusion injury by reducing reactive oxygen species signaling and reducing neuronal apoptosis in the hippocampus by blocking the opening of mitochondrial permeability transition pore (mPTP) [27, 28]. In addition, recent studies have reported that pharmacology postconditioning and ischemia preconditioning attenuated ischemia reperfusion injury via the necroptosis pathway [26, 29]. Volatile anesthetic postconditioning protects against ischemia reperfusion injury by regulating the calcium/calmodulin-dependent protein kinase II (CaMKII). Sustained CaMKII activation has been identified as a central mediator of necroptosis in cardiovascular diseases [30, 31].

Necroptosis plays a major role in myocardial infarction remodeling, heart failure, cardiac dysfunction, and disease progression [32–34]. Necroptosis is a form of regulated cell death that is regulated by RIPK3 and MLKL. Necroptosis generally manifests with morphological features similar to necrosis [35]. Necroptosis was shown to be dependent on the RIPK1-RIPK3-MLKL signaling pathway in ischemia reperfusion hearts [36–38]. Studies have demonstrated that Necrostatin-1 prevents necroptosis after brain ischemic stroke by inhibiting RIPK1-mediated RIPK3/MLKL signaling. microRNA-325-3p has been shown to protect the heart against ischemia reperfusion injury by inhibiting RIPK3 and necroptosis in mouse models [39, 40]. In this study, we measured the levels of serum cardiac necroptosis marker enzyme LDH using Evans blue dye uptake, as well as the expression levels of important regulatory proteins of necroptosis. Our results demonstrated that SPC administration reduced LDH levels and RIPK3/MLKL mediated necroptosis induced by MIRI. This strongly suggested the cardioprotective effects of SPC. These results were consistent with previous studies that demonstrated that OGT deficient cells underwent excessive necroptosis and exhibited elevated protein expression levels of RIPK3 and MLKL, which are key mediators of necroptosis. O-GlcNAc transferase suppresses inflammation and necroptosis by targeting RIPK3 [8, 12]. Previous studies have reported that isoflurane-induced cardiac protection against ischemia reperfusion injury was via O-GlcNAc modification of mitochondrial voltage-dependent anion channels [15].

O-GlcNAc plays a significant role in cellular function and multiple diseases. O-GlcNAc increases in response to stress and has been shown to be protective against ischemia reperfusion injury. Human, animal, and laboratory studies have demonstrated that ischemic preconditioning protects against ischemia reperfusion injury and is associated with an increase in O-GlcNAc levels [41]. In addition, glucosamine was shown to induce neuroprotective effects against IR injury by increasing cerebral O-GlcNAc levels [42]. In our study, O-GlcNAc, OGT, and OGA protein expression levels in the IR group were increased compared to the SHAM group. This may be an endogenous protective response. Our *in vivo* experiments demonstrated that SPC administration further increased OGT mediated O-GlcNAcylation, enabled OGT-mediated O-GlcNAcylation of RIPK3, and reduced the formation of the RIPK3/MLKL complex. SPC induced cardioprotective effects may be associated with OGT mediated O-GlcNAcylation. To further clarify the effect of OGT mediated O-GlcNAcylation in SPC mediated cardioprotection, an OGT inhibitor OSMI-1 was used in our *in vitro* studies. We demonstrated that OSMI-1 inhibited OGT mediated O-GlcNAcylation and increased necroptosis to reverse the cardioprotective effects induced by SPC.

## CONCLUSIONS

In summary, we were the first to demonstrate that SPC restrained cardiomyocytes necroptosis via regulating OGT mediated O-GlcNAcylation of RIPK3 and lessening RIPK3/MLKL induced necroptosis, and hence protected the heart against MIRI.

Limitations of our study included the following: we did not determine whether SPC promotes RIPK3 degradation by directly affecting OGT. Our future studies will address this mechanism. In a word, we demonstrated that SPC administration had a cardioprotective role on MIRI. The protective mechanism of SPC was via the activation of OGT resulting in O-GlcNAcylation of RIPK3 to reduce RIPK3/MLKL mediated necroptosis.

## MATERIALS AND METHODS

### Animals

A total of 105 healthy male Sprague-Dawley rats, weighing 180-230 g were included in this study. All rats were kept in individual cages with a 12-hour light, 12-hour dark cycle at 22-24° C. The rats were allowed free access to food and water, and both sexes were included in the experiments. All the experiments in this study were approved by the Institutional Animal Care and Use Committee of Nanchang University and performed in accordance with the guidelines for the Principles of Laboratory Animal Care and Use of Laboratory Animals published by NIH (NIH Publication, 8^th^ Edition, 2011).

### MIRI *in vivo* surgical preparation

Coronary artery ligation method was used to establish MIRI *in vivo* model [43]. Rats were anaesthetized with sodium pentobarbital (50 mg/kg) and heparinized (1000 IU/kg) by intraperitoneal injection to ensure that pedal and palpebral reflexes were absent throughout the experimental protocol. The rats received endotracheal intubation and artificial ventilation under electrocardiogram equipment (GE Medical, Milwaukee, WI, USA) monitoring. A thoracotomy was performed in the left fifth intercostal space, and the pericardium was opened. Then, a 6-0 silk suture was placed around the proximal left anterior descending (LAD) appendage for 2-3 mm through a small polytetra fluoroethylene tube, which formed a snare. LAD occlusion was produced by pulling the snare for 30 min, while successful reperfusion was achieved by loosening the snare for 2 h. A polyethylene catheter placed into the left ventricle and connecting a pressure transducer to a data acquisition system (Medlab-U/4C501H system) was used to measure hemodynamic parameters (heart rate, HR; mean arterial blood pressure, MAP; rate pressure product, RPP). And they were recorded at 30 min of equilibration (T_0_), 30 min (T_1_), 60 min (T_2_), 90 min (T_3_), and 2 h (T_4_) after reperfusion. Successful occlusion and subsequent development of ischemia were confirmed by prompt ST-segment changes on ECG with progressive ST-segment elevation in at least three leads with or without arrythmia, and decolorization of the myocardium distal to the occlusion. Moreover, reperfusion and restoration of blood flow were confirmed by prompt ST-segment changes on ECG with progressive ST-segment normalization/depression and pathological Q-wave formation in leads with previous ST-segment elevation with or without arrythmia, and re-colorization of the affected myocardium [44].

### Langendorff isolated heart perfusion model

Anesthetization and preparation of isolated hearts were performed as described previously with slight modification [45]. In brief, rats were anaesthetized and then the rat hearts were quickly excised and mounted on a modified non-circulating Langendorff apparatus via aorta cannulation for retrograde perfusion at constant pressure (80 mmHg) with Krebs-Henseleit (K-H) buffer (solution configuration: NaCl 118.0 mmol/L, KCl 4.8 mmol/L, KH2PSO4 1.2 mmol/L, NaHCO3 25.0 mmol/L, MgSO4 1.2 mmol/L, CaCl2 2.5 mmol/L, glucose 11.0 mmol/L, and pH 7.35-7.45). The K-H buffer was gassed with 95% O2-5% CO2, and the temperature was maintained at 37C. And, a small latex balloon connected to a cuff pressure transducer (SIA Industrial & Trade, Beijing, China) was inserted into the left ventricle through the mitral valve to monitor the rat hemodynamics indexes (HR; left ventricular peak pressure, LVSP; left ventricular end diastolic pressure, LVEDP; maximal rate of the increase/decrease of left ventricular pressure, ±dp/dt_max_). Hemodynamics indexes were recorded at T_0_, T_1_, T_2_, T_3_ and T_4_.

### *In vivo* IR model and Isolated IR model

**Figure 8:**
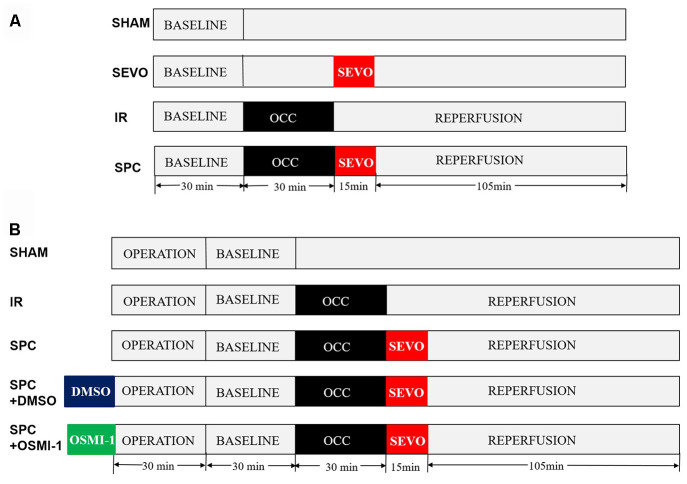
**Schematic illustration of the experimental protocol *in vivo* and *in vitro*.** (**A**) All the groups underwent the same surgical operation. (1) SHAM: rats were subjected to open chest surgery only; (2) SEVO: rats received 1.0 MAC sevoflurane for 15 min without occlusion; (3) I/R: rats were subjected to 30 min LAD occlusion, followed by 2 h of reperfusion; (4) SPC: rats were subjected to I/R and receiving 1.0MAC sevoflurane for 15 min at onset of reperfusion. (**B**) Except the SHAM group, all hearts was subjected to 30 min of global ischemia, followed by 2 h of reperfusion. SPC group received 1.0 MAC sevoflurane for 15 min at onset of reperfusion. In the SPC+DMSO group or SPC+OSMI-1 group, in addition to SPC administration, 50 μM DMSO or OSMI-1 was given and present throughout the experiment.

Rats were randomly divided into four groups: SHAM group, SEVO group, IR group and SPC group. Except the SHAM and SEVO groups, each rat was subjected to 30 min of LAD occlusion, followed by 2 h of reperfusion. In the SEVO group, the rats received 1.0 minimum alveolar concentration (MAC) sevoflurane (2.4% sevoflurane, 37° C) for 15 min without occlusion. Sevoflurane (Maruishi Pharmaceutical Co, Chuoku, Osaka, Japan) was added to the inspired gas starting 1 min before reperfusion to achieve the concentration at 1.0 MAC. In the SPC group, after 30 min of LAD occlusion, the rats inhaled 1.0 MAC sevoflurane for 15 min starting from the end of ischemia until 15 min after reperfusion, then reperfusion the hearts for 105 min. The experimental design is shown in Figure 8A.

Isolated perfused rat hearts were randomly divided into five groups: SHAM group, IR group, SPC group, SPC+DMSO group and SPC+OSMI-1 group. Except the SHAM group, each rat was subjected to 30 min of global ischemia, followed by 2 h of reperfusion. In SPC group, the hearts were perfused with K-H solution saturated with 1.0 MAC sevoflurane for 15 min starting from the end of ischemia until 15 min after reperfusion, and then with plain K-H solution for 105 min. SPC was administrated as previously described [46]. In brief, sevoflurane was bubbled into the K-H solution by using an agent specific vaporizer (Dräger Medizintechnik GmbH, Lübeck, Germany) placed in the 95%O2-5%CO2 gas mixtures line at a concentration of (1.2±0.02) mmol/L (8%, v/v) measured in the liquid phase by a datex infrared gas analyzer (Ohmeda, GE Healthcare), and ensure that the concentration of delivered sevoflurane in the K-H solution was maintained at 1.0 MAC. Moreover, in the SPC+OSMI-1 group or SPC+DMSO group, in addition to SPC administration, 50 μM OSMI-1 (the OGT inhibitor, Abcam Co., Cambridgeshire, UK) or the OSMI-1 solvent DMSO was given in the K-H solution and present throughout the experiment. The experimental design is shown in Figure 8B.

### Myocardial infarction size measurement

Myocardial infarction size was evaluated by 2,3,5-triphenyltetrazolium chloride triazole (TTC; Sigma Aldrich Co., St. Louis, MO, USA, 1% in PBS) staining [47]. At the end of 2 h reperfusion, the rat hearts were frozen at -20° C and then cut into five pieces in cross-section. The hearts were incubated in 1% TTC (pH 7.4) at 37° C for 10 min, and subsequently fixed overnight in 10% formalin. The area of infarcted tissues (white or pale color) could be differentiated from the area at risk (red color). The infarct size was calculated for each slice, and reported as the percent of infarct divided by the total area at risk by Alpha Ease FC Imaging System.

### Echocardiography evaluation

After rats were anaesthetized with sodium pentobarbital, two-dimensional echocardiography measurements were conducted at the end of reperfusion. The structure and heart function of each subject were evaluated by M-mode echocardiography using the Vevo770 system equipped with a 35-MHz linear transducer as previous methods [48]. Three consecutive cardiac cycles were captured by digital image analysis software. The measurement was operated by an independent professional ultrasound technician, and the independent professional echocardiographer was blinded to the groups. Parameters of cardiac function were obtained in the M-mode tracings. Moreover, ejection fraction (EF)% and fractional shortening (FS)% of the left ventricle were calculated according to standard formulas: EF% = (LVEDd^3^-LVESd^3^)×100%/LVEDd^3^, where LVEDd is left ventricular end-diastolic diameter, LVESd is left ventricular end-systolic diameter; FS% = (LVEDd-LVESd)×100%/LVEDd. And EF, FS and stroke volume (SV) were automatic calculated by Vevo770 Visual sonics software.

### Myocardial LDH content measurement

After the 2h reperfusion, serum samples were isolated from the blood samples by centrifugation at 3000 rpm, 4° C, for 10 min. The level of serum cardiac marker enzyme lactic dehydrogenase (LDH) was measured using commercially available LDH kits (Nanjing Jiancheng Bioengineering Institute, Nanjing, China) according to the manufacturer’s instruction.

### Histology

After the 2h reperfusion, the hearts tissue was immediately removed, fixed in 10% formalin and then embedded in paraffin. The staining of hematoxylin-eosin (H&E) was performed on 3-5μm sections of cardiac tissue cut from the 10% formaldehyde solution-fixed, paraffin-embedded blocks [49] (n = 3/group). The histopathological changes were examined under an optical microscope (Leica, Germany).

### Myocardium tissue necrosis staining

As previously described, the degree of MIRI was assessed using Evans blue (Sigma Aldrich Co., St. Louis, MO, USA) staining [50]. In brief, rats were given with Evans blue dye (final concentration of 1% volume to body weight) through tail vein injection before sacrifice. At the end of reperfusion, all heart tissues were taken and cut into 5μm cryosections to measure the amount of Evans blue dye uptake (red autofluorescence) by fluorescence microscopy equipped to acquire fluorescence images with a light microscope using 200× magnifications. And, EBD-positive areas are expressed as the percent area of the myocardium with red fluorescence, and all data were determined with Scion Image software.

### Co-immunoprecipitation

Co-immunoprecipitation (Co-IP) analysis was as described previously [51]. The heart tissues were homogenate in modified RIPA buffer by sonication and frozen on dry ice for 60 min. Residual myocardial debris was removed by centrifugation at 18,000 × g, 4° C for 15 min. Protein concentration was measured, 50 μg of total protein was used for immunoprecipitation, 25 μg was used as an input control. Then, each IP reaction was adjusted to a total volume of 300 μl in microfuge tubes by the addition of lysis buffer containing protease and phosphatase inhibitors. 2 μl of anti-O-GlcNAc antibody (RL2) or anti-RIPK3 antibody (Santa Cruz Co., California, USA) was added. Tubes were incubated for approximately 15 h at 4° C on a rotor. Subsequently, 50 μl of a 50:50 slurry of protein A/G plus-agarose beads (Santa Cruz Co., California, USA) was added, followed by incubation at 4° C overnight. The beads were resuspended in LDS buffer that contained 50 mmol/L DTT. Finally, the resulting co-IP reaction and input controls were detected by western blotting with anti-RIPK3 (1:1000 dilution, Santa Cruz Co., California, USA), anti-RL2 (1:2000 dilution, Santa Cruz Co., California, USA) and anti-MLKL (1:1000 dilution, Santa Cruz Co., California, USA).

### Western blot analysis

Western blot analysis was performed as described previously [52]. Briefly, after the 2h reperfusion, cardiac tissue samples were collected and cellular protein extracts were prepared. The concentrations of proteins were determined using a bicinchoninic acid protein assay kit (Thermo Fisher Co., Massachusetts, USA). Equivalent amount of proteins (30 mg) were separated by 12% SDS-polyacrylamide gel electrophoresis and then transferred onto a polyvinylidene fluoride (PVDF) membrane. After blocking with 5% non-fat milk at room temperature for 1 h, primary antibodies against p-RIPK1, RIPK1, p-RIPK3, RIPK3, p-MLKL, MLKL (1:1000 dilution, Thermo Fisher Co., Massachusetts, USA), O-GlcNAc, OGT, OGA (1:1000 dilution, Santa Cruz Co., California, USA) were incubated with the PVDF membranes at 4° C overnight. Horseradish peroxidase-conjugated mouse anti-rabbit IgG secondary antibody (1:2,000 dilution, Cell Signaling Technology Co., Colorado, USA) were incubated with the membranes at room temperature for 1 h. The signals were visualized with enhanced chemiluminescence western blotting detection system (GE Healthcare, Chicago, IL, USA). To control for lane loading, the same membranes were probed with anti-GAPDH (1:5000 dilution, Beyotime Institute of Biotechnology, Shanghai, China). Quantitative analysis of the signals was performed by scanning densitometry and analyzed using Image Lab Software (version 4.1; Bio-Rad Laboratories, Inc.).

### Statistical analysis

The results are shown as means ± standard deviation. All experimental data were analyzed by using one-way analysis of variance (ANOVA) followed by Tukey multiple comparison test. P<0.05 was considered to be statistically significant difference.

## References

[1] Davidson SM, Ferdinandy P, Andreadou I, Bøtker HE, Heusch G, Ibáñez B, Ovize M, Schulz R, Yellon DM, Hausenloy DJ, Garcia-Dorado D, and CARDIOPROTECTION COST Action (CA16225). Multitarget strategies to reduce myocardial ischemia/reperfusion injury: JACC review topic of the week. J Am Coll Cardiol. 2019; 73:89–99. 10.1016/j.jacc.2018.09.086 30621955

[2] Palanca BJ, Avidan MS, Mashour GA. Human neural correlates of sevoflurane-induced unconsciousness. Br J Anaesth. 2017; 119:573–82. 10.1093/bja/aex244 29121298 PMC6172973

[3] Zhang J, Wang C, Yu S, Luo Z, Chen Y, Liu Q, Hua F, Xu G, Yu P. Sevoflurane postconditioning protects rat hearts against ischemia-reperfusion injury via the activation of PI3K/AKT/mTOR signaling. Sci Rep. 2014; 4:7317. 10.1038/srep07317 25471136 PMC4255182

[4] Yu P, Zhang J, Yu S, Luo Z, Hua F, Yuan L, Zhou Z, Liu Q, Du X, Chen S, Zhang L, Xu G. Protective effect of sevoflurane postconditioning against cardiac ischemia/reperfusion injury via ameliorating mitochondrial impairment, oxidative stress and rescuing autophagic clearance. PLoS One. 2015; 10:e0134666. 10.1371/journal.pone.0134666 26263161 PMC4532466

[5] Wang P, Shao BZ, Deng Z, Chen S, Yue Z, Miao CY. Autophagy in ischemic stroke. Prog Neurobiol. 2018; 163–164:98–117. 10.1016/j.pneurobio.2018.01.001 29331396

[6] Szobi A, Farkašová-Ledvényiová V, Lichý M, Muráriková M, Čarnická S, Ravingerová T, Adameová A. Cardioprotection of ischaemic preconditioning is associated with inhibition of translocation of MLKL within the plasma membrane. J Cell Mol Med. 2018; 22:4183–96. 10.1111/jcmm.13697 29921042 PMC6111849

[7] Galluzzi L, Vitale I, Aaronson SA, Abrams JM, Adam D, Agostinis P, Alnemri ES, Altucci L, Amelio I, Andrews DW, Annicchiarico-Petruzzelli M, Antonov AV, Arama E, et al. Molecular mechanisms of cell death: recommendations of the Nomenclature Committee on cell death 2018. Cell Death Differ. 2018; 25:486–541. 10.1038/s41418-017-0012-4 29362479 PMC5864239

[8] Li X, Gong W, Wang H, Li T, Attri KS, Lewis RE, Kalil AC, Bhinderwala F, Powers R, Yin G, Herring LE, Asara JM, Lei YL, et al. O-GlcNAc Transferase Suppresses Inflammation and Necroptosis by Targeting Receptor-Interacting Serine/Threonine-Protein Kinase 3. Immunity. 2019; 50:576–590.e6. 10.1016/j.immuni.2019.01.007 30770249 PMC6426684

[9] Torres CR, Hart GW. Topography and polypeptide distribution of terminal N-acetylglucosamine residues on the surfaces of intact lymphocytes. Evidence for O-linked GlcNAc. J Biol Chem. 1984; 259:3308–17. 6421821

[10] Yang X, Qian K. Protein O-GlcNAcylation: emerging mechanisms and functions. Nat Rev Mol Cell Biol. 2017; 18:452–65. 10.1038/nrm.2017.22 28488703 PMC5667541

[11] Hart GW, Housley MP, Slawson C. Cycling of O-linked beta-N-acetylglucosamine on nucleocytoplasmic proteins. Nature. 2007; 446:1017–22. 10.1038/nature05815 17460662

[12] Zhang B, Li MD, Yin R, Liu Y, Yang Y, Mitchell-Richards KA, Nam JH, Li R, Wang L, Iwakiri Y, Chung D, Robert ME, Ehrlich BE, et al. O-GlcNAc transferase suppresses necroptosis and liver fibrosis. JCI Insight. 2019; 4:e127709. 10.1172/jci.insight.127709 31672932 PMC6948774

[13] Zachara NE, O’Donnell N, Cheung WD, Mercer JJ, Marth JD, Hart GW. Dynamic O-GlcNAc modification of nucleocytoplasmic proteins in response to stress. A survival response of mammalian cells. J Biol Chem. 2004; 279:30133–42. 10.1074/jbc.M403773200 15138254

[14] Martinez MR, Dias TB, Natov PS, Zachara NE. Stress-induced O-GlcNAcylation: an adaptive process of injured cells. Biochem Soc Trans. 2017; 45:237–49. 10.1042/BST20160153 28202678 PMC6492270

[15] Hirose K, Tsutsumi YM, Tsutsumi R, Shono M, Katayama E, Kinoshita M, Tanaka K, Oshita S. Role of the O-linked β-N-acetylglucosamine in the cardioprotection induced by isoflurane. Anesthesiology. 2011; 115:955–62. 10.1097/ALN.0b013e31822fcede 21876430

[16] Miller DL, Li P, Dou C, Armstrong WF, Gordon D. Evans blue staining of cardiomyocytes induced by myocardial contrast echocardiography in rats: evidence for necrosis instead of apoptosis. Ultrasound Med Biol. 2007; 33:1988–96. 10.1016/j.ultrasmedbio.2007.06.008 17689176 PMC2204068

[17] Giogha C, Lawlor KE. Sugar fix keeps RIPK3 at bay. Immunity. 2019; 50:539–41. 10.1016/j.immuni.2019.02.018 30893581

[18] Jaskiewicz NM, Townson DH. Hyper-O-GlcNAcylation promotes epithelial-mesenchymal transition in endometrial cancer cells. Oncotarget. 2019; 10:2899–910. 10.18632/oncotarget.26884 31080560 PMC6499600

[19] Grievink H, Kuzmina N, Chevion M, Drenger B. Sevoflurane postconditioning is not mediated by ferritin accumulation and cannot be rescued by simvastatin in isolated streptozotocin-induced diabetic rat hearts. PLoS One. 2019; 14:e0211238. 10.1371/journal.pone.0211238 30682140 PMC6347357

[20] Li H, Chen D, Fang N, Yao Y, Li L. Age-associated differences in response to sevoflurane postconditioning in rats. Scand Cardiovasc J. 2016; 50:128–36. 10.3109/14017431.2015.1122830 26667494

[21] Grishin AV, Yavorovskiy AG, Charchian ER, Fedulova SV, Chamaia MA. [Pharmacological postconditioning by sevoflurane during cardiac surgery]. Anesteziol Reanimatol. 2016; 61:348–52. 29489101

[22] Kim HC, Kim E, Bae JI, Lee KH, Jeon YT, Hwang JW, Lim YJ, Min SW, Park HP. Sevoflurane Postconditioning Reduces Apoptosis by Activating the JAK-STAT Pathway After Transient Global Cerebral Ischemia in Rats. J Neurosurg Anesthesiol. 2017; 29:37–45. 10.1097/ANA.0000000000000331 27337676

[23] Shiraishi S, Cho S, Akiyama D, Ichinomiya T, Shibata I, Yoshitomi O, Maekawa T, Ozawa E, Miyaaki H, Hara T. Sevoflurane has postconditioning as well as preconditioning properties against hepatic warm ischemia-reperfusion injury in rats. J Anesth. 2019; 33:390–98. 10.1007/s00540-019-02642-4 31053929

[24] Hu X, Wang J, Zhang L, Zhang Q, Duan X, Zhang Y. Postconditioning with sevoflurane ameliorates spatial learning and memory deficit via attenuating endoplasmic reticulum stress induced neuron apoptosis in a rat model of hemorrhage shock and resuscitation. Brain Res. 2018; 1696:49–55. 10.1016/j.brainres.2018.05.047 29870695

[25] Ohsumi A, Marseu K, Slinger P, McRae K, Kim H, Guan Z, Hwang DM, Liu M, Keshavjee S, Cypel M. Sevoflurane attenuates ischemia-reperfusion injury in a rat lung transplantation model. Ann Thorac Surg. 2017; 103:1578–86. 10.1016/j.athoracsur.2016.10.062 28190546

[26] Miklić Bublić M, Tonković D, Sakan S, Misir A, Bandić Pavlović D. Effect of inhalational anesthetics on acute kidney injury. Acta Clin Croat. 2016; 55:464–68. 10.20471/acc.2016.55.03.16 29045774

[27] Beck-Schimmer B, Roth Z’graggen B, Booy C, Köppel S, Spahn DR, Schläpfer M, Schadde E. Sevoflurane protects hepatocytes from ischemic injury by reducing reactive oxygen species signaling of hepatic stellate cells: translational findings based on a clinical trial. Anesth Analg. 2018; 127:1058–65. 10.1213/ANE.0000000000003692 30216289

[28] Zhang L, Huang L, Wang J, Zhang M, Zhang Y, Hu X. Sevoflurane postconditioning improves spatial learning and memory ability involving mitochondrial permeability transition pore in hemorrhagic shock and resuscitation rats. Brain Behav. 2020; 10:e01501. 10.1002/brb3.1501 31833229 PMC6955830

[29] Dong XH, Liu H, Zhang MZ, Zhao PX, Liu S, Hao Y, Wang YB. Postconditioning with inhaled hydrogen attenuates skin ischemia/reperfusion injury through the RIP-MLKL-PGAM5/Drp1 necrotic pathway. Am J Transl Res. 2019; 11:499–508. 30788005 PMC6357323

[30] McMurtrey RJ, Zuo Z. Isoflurane preconditioning and postconditioning in rat hippocampal neurons. Brain Res. 2010; 1358:184–90. 10.1016/j.brainres.2010.08.015 20709037 PMC2949531

[31] Reventun P, Sanchez-Esteban S, Cook A, Cuadrado I, Roza C, Moreno-Gomez-Toledano R, Muñoz C, Zaragoza C, Bosch RJ, Saura M. Bisphenol a induces coronary endothelial cell necroptosis by activating RIP3/CamKII dependent pathway. Sci Rep. 2020; 10:4190. 10.1038/s41598-020-61014-1 32144343 PMC7060177

[32] Luedde M, Lutz M, Carter N, Sosna J, Jacoby C, Vucur M, Gautheron J, Roderburg C, Borg N, Reisinger F, Hippe HJ, Linkermann A, Wolf MJ, et al. RIP3, a kinase promoting necroptotic cell death, mediates adverse remodelling after myocardial infarction. Cardiovasc Res. 2014; 103:206–16. 10.1093/cvr/cvu146 24920296

[33] Szobi A, Gonçalvesová E, Varga ZV, Leszek P, Kuśmierczyk M, Hulman M, Kyselovič J, Ferdinandy P, Adameová A. Analysis of necroptotic proteins in failing human hearts. J Transl Med. 2017; 15:86. 10.1186/s12967-017-1189-5 28454582 PMC5410070

[34] Zhang T, Zhang Y, Cui M, Jin L, Wang Y, Lv F, Liu Y, Zheng W, Shang H, Zhang J, Zhang M, Wu H, Guo J, et al. CaMKII is a RIP3 substrate mediating ischemia- and oxidative stress-induced myocardial necroptosis. Nat Med. 2016; 22:175–82. 10.1038/nm.4017 26726877

[35] Galluzzi L, Kepp O, Chan FK, Kroemer G. Necroptosis: mechanisms and relevance to disease. Annu Rev Pathol. 2017; 12:103–30. 10.1146/annurev-pathol-052016-100247 27959630 PMC5786374

[36] Qin D, Wang X, Li Y, Yang L, Wang R, Peng J, Essandoh K, Mu X, Peng T, Han Q, Yu KJ, Fan GC. MicroRNA-223-5p and -3p cooperatively suppress necroptosis in ischemic/reperfused hearts. J Biol Chem. 2016; 291:20247–59. 10.1074/jbc.M116.732735 27502281 PMC5025706

[37] Koshinuma S, Miyamae M, Kaneda K, Kotani J, Figueredo VM. Combination of necroptosis and apoptosis inhibition enhances cardioprotection against myocardial ischemia-reperfusion injury. J Anesth. 2014; 28:235–41. 10.1007/s00540-013-1716-3 24113863

[38] Oerlemans MI, Liu J, Arslan F, den Ouden K, van Middelaar BJ, Doevendans PA, Sluijter JP. Inhibition of RIP1-dependent necrosis prevents adverse cardiac remodeling after myocardial ischemia-reperfusion *in vivo*. Basic Res Cardiol. 2012; 107:270. 10.1007/s00395-012-0270-8 22553001

[39] Deng XX, Li SS, Sun FY. Necrostatin-1 prevents necroptosis in brains after ischemic stroke via inhibition of RIPK1-mediated RIPK3/MLKL signaling. Aging Dis. 2019; 10:807–17. 10.14336/AD.2018.0728 31440386 PMC6675533

[40] Zhang DY, Wang BJ, Ma M, Yu K, Zhang Q, Zhang XW. MicroRNA-325-3p protects the heart after myocardial infarction by inhibiting RIPK3 and programmed necrosis in mice. BMC Mol Biol. 2019; 20:17. 10.1186/s12867-019-0133-z 31248365 PMC6598367

[41] Jensen RV, Andreadou I, Hausenloy DJ, Bøtker HE. The role of O-GlcNAcylation for protection against ischemia-reperfusion injury. Int J Mol Sci. 2019; 20:404. 10.3390/ijms20020404 30669312 PMC6359045

[42] Hwang SY, Shin JH, Hwang JS, Kim SY, Shin JA, Oh ES, Oh S, Kim JB, Lee JK, Han IO. Glucosamine exerts a neuroprotective effect via suppression of inflammation in rat brain ischemia/reperfusion injury. Glia. 2010; 58:1881–92. 10.1002/glia.21058 20737476

[43] Bi X, Zhang G, Wang X, Nguyen C, May HI, Li X, Al-Hashimi AA, Austin RC, Gillette TG, Fu G, Wang ZV, Hill JA. Endoplasmic reticulum chaperone GRP78 protects heart from ischemia/reperfusion injury through Akt activation. Circ Res. 2018; 122:1545–54. 10.1161/CIRCRESAHA.117.312641 29669712 PMC5970094

[44] Sayour AA, Korkmaz-Icöz S, Loganathan S, Ruppert M, Sayour VN, Oláh A, Benke K, Brune M, Benkő R, Horváth EM, Karck M, Merkely B, Radovits T, Szabó G. Acute canagliflozin treatment protects against *in vivo* myocardial ischemia-reperfusion injury in non-diabetic male rats and enhances endothelium-dependent vasorelaxation. J Transl Med. 2019; 17:127. 10.1186/s12967-019-1881-8 30992077 PMC6469222

[45] Karam S, Margaria JP, Bourcier A, Mika D, Varin A, Bedioune I, Lindner M, Bouadjel K, Dessillons M, Gaudin F, Lefebvre F, Mateo P, Lechène P, et al. Cardiac overexpression of PDE4B blunts β-adrenergic response and maladaptive remodeling in heart failure. Circulation. 2020; 142:161–74. 10.1161/CIRCULATIONAHA.119.042573 32264695

[46] Qiao SG, Sun Y, Sun B, Wang A, Qiu J, Hong L, An JZ, Wang C, Zhang HL. Sevoflurane postconditioning protects against myocardial ischemia/reperfusion injury by restoring autophagic flux via an NO-dependent mechanism. Acta Pharmacol Sin. 2019; 40:35–45. 10.1038/s41401-018-0066-y 30002490 PMC6318323

[47] Zhang J, Zhang J, Yu P, Chen M, Peng Q, Wang Z, Dong N. Remote ischaemic preconditioning and sevoflurane postconditioning synergistically protect rats from myocardial injury induced by ischemia and reperfusion partly via inhibition TLR4/MyD88/NF-κB signaling pathway. Cell Physiol Biochem. 2017; 41:22–32. 10.1159/000455815 28135708

[48] Khalique OK, Cavalcante JL, Shah D, Guta AC, Zhan Y, Piazza N, Muraru D. Multimodality imaging of the tricuspid valve and right heart anatomy. JACC Cardiovasc Imaging. 2019; 12:516–31. 10.1016/j.jcmg.2019.01.006 30846125

[49] Varasteh Z, Mohanta S, Robu S, Braeuer M, Li Y, Omidvari N, Topping G, Sun T, Nekolla SG, Richter A, Weber C, Habenicht A, Haberkorn UA, Weber WA. Molecular Imaging of Fibroblast Activity After Myocardial Infarction Using a ^68^Ga-Labeled Fibroblast Activation Protein Inhibitor, FAPI-04. J Nucl Med. 2019; 60:1743–1749. 10.2967/jnumed.119.226993 31405922 PMC6894377

[50] Sachdeva J, Dai W, Gerczuk PZ, Kloner RA. Combined remote perconditioning and postconditioning failed to attenuate infarct size and contractile dysfunction in a rat model of coronary artery occlusion. J Cardiovasc Pharmacol Ther. 2014; 19:567–73. 10.1177/1074248413518967 24607766

[51] Morrison A, Chen L, Wang J, Zhang M, Yang H, Ma Y, Budanov A, Lee JH, Karin M, Li J. Sestrin2 promotes LKB1-mediated AMPK activation in the ischemic heart. FASEB J. 2015; 29:408–17. 10.1096/fj.14-258814 25366347 PMC4314228

[52] Badolia R, Ramadurai DK, Abel ED, Ferrin P, Taleb I, Shankar TS, Krokidi AT, Navankasattusas S, McKellar SH, Yin M, Kfoury AG, Wever-Pinzon O, Fang JC, et al. The role of nonglycolytic glucose metabolism in myocardial recovery upon mechanical unloading and circulatory support in chronic heart failure. Circulation. 2020; 142:259–74. 10.1161/CIRCULATIONAHA.119.044452 32351122 PMC7380956

